# Isolation of functional ligninolytic *Bacillus aryabhattai* from paper mill sludge and its lignin degradation potential

**DOI:** 10.1016/j.btre.2022.e00755

**Published:** 2022-07-18

**Authors:** Anjali Singh, Rajesh Kumar, Annapurna Maurya, Pankaj Chowdhary, Abhay Raj

**Affiliations:** a Environmental Microbiology Laboratory, Environmental Toxicology Group, CSIR-Indian Institute of Toxicology Research (CSIR-IITR), Lucknow 226001, Uttar Pradesh, India; b Academy of Scientific and Innovative Research (AcSIR), Ghaziabad 201002, Uttar Pradesh, India

**Keywords:** Kraft lignin, Bacillus aryabhattai, Biodegradation, SEM, FTIR, GC–MS

## Abstract

Kraft lignin (KL), is the major pollutant in pulp and paper effluent and due to its heterogeneous structure, it is resistant to the depolymerization process. It has drawn much attention from the researcher due to its challenging degradation process. In this study, a KL-degrading bacterium was isolated and screened from paper mill sludge. This bacterium was identified as ligninolytic *Bacillus aryabhattai* using biochemical and 16SrRNA gene analysis. *B. aryabhattai* showed maximum activities of lignin peroxidase-LiP (0.74 IU mL^−1^) and manganese peroxidase-MnP (9.2 IU mL^−1^) on the 4th day, and 5th day, respectively. A total 84% of KL (500 mg *L*^−1^) reduction was observed after 14 days. The KL bio-degradation was confirmed based on changes in chemical stracture of KL and new metabolites identification using FTIR and GC–MS, respectively. The study concluded that *B. aryabhattai* maybe becomes a potential biological agent in KL biodegradation and treatment of other lignin-containing industrial effluents.

## Highlights

• Isolation of a functional lignin-degrading *Bacillus aryabhattai*.
• Production of growth-associated LiP and MnP enzymes.
• Almost 84% KL degradation at 500 mg *L*^−1^ KL concentration.
• KL biodegradation process was revealed by chemical analysis.

## 1 Introduction

The total land-based biomass comprises about 25% heterogeneous aromatic polymer lignin. It is composed of phenylpropanoid units i.e. guaiacyl units, p-hydroxyphenyl units, and syringyl units, from respective precursor molecules that make them highly stable [15]. However, KL is a type of industrial lignin that is formed during the pulping of the paper-making process. KL has been considered as inherent heterogeneity and recalcitrance due to the intricate chemical bonding among monomers [13]. Globally, the pulp and paper industries generate ∼50 million tons of lignin annually and out of which ∼2% recuperated for the chemical manufacturing application [10,20]. Improper treatments of KL containing industrial waste make them the main environmental polluter [28]. The pulp and paper industry discharged effluent with high characteristic as dark brown color with alkaline (generally) pH, high chemical oxygen demand (1110–1272 mg L^−1^), suspended solids (1160–1380 mg L^−1^), dissolved solids (1043–129 mg L^−1^) and lignin contents [34]. Once it enters the aquatic ecosystem, it reduces the level of dissolved oxygen and also hinders the photosynthesis process, which adversely affects the flora and fauna [31].

The KL degradation technologies are physical (membrane filtration, sedimentation), chemical (chemical oxidation and ozonation), and biological-based (activated sludge, anaerobic-aerobic treatment, membrane bioreactor) [30]. During physical processes, operational costs and energy consumption are very high and chemical processes are responsible for the generation of secondary pollutants from the mismanagement of the chemicals used. On the contrary, the bioremediation approach that uses fungi and bacteria that have the potential to evolve continuously in nature to degrade KL is an environment-friendly manner and needs relatively less energy consumption during the KL degradation [22]. Additionally, the degradation of ligninocellusic biomass *via* fungi has been comprehensively studied [9]. However, due to their sensitivity to physiological environmental conditions, i.e. temperature and pH, fungi are not fulfilling the industrial demand for lignocellulosic waste treatment [23]. Unlikely fungal cells, bacteria are specified by a higher growth rate with a wider tolerance range (pH and temperature), a synergy among complex enzymatic systems, and higher feasibility toward genetic engineering approaches [2]. Recent studies have mentioned the potential involvement of different bacterial species like *B. ligniniphilus, Rhodococcus jostii, Pandoraea* sp., and sulfate-reducing bacteria in lignin degradation and pre-treatment of lignocellulosic biomass [37,44,45]. KL degrading bacteria belong to three different classes, which are as follows: (i) α-proteobacteria, (ii) γ- proteobacteria, and (iii) actinomycetes; these were isolated from the various sources such as soil, sediments, animals, insect guts, etc. [19]. However, still, there are some other microbes with specific properties, which can degrade or decompose the KL more efficiently, which need to be identified and explored in detail. Laccase, LiP, and MnP are major ligninolytic enzymes, which have attracted more attention in the recent decade for the decay/breakdown of lignocellulosic waste and their derivatives [7]. Furthermore, laccase is a copper-containing enzyme while LiP and MnP are heme-containing enzymes produced by various microorganisms that retain the potential to degrade KL [24].

Therefore, this study aims to investigate the KL degradation process using the ligninolytic bacteria (*B. aryabhattai)* isolated from paper mill sludge. Besides, the SEM, FTIR, and GC–MS analyses were performed to analyze the KL degradation process. This study provides an important basis for lignin degradation by identified bacterial strains and it may be used in treatment of lignin-containing industrial effluents with divers industrial biotechnological applications.

## 2 Materials and methods

### 2.1 Materials

All chemicals, reagents, solvents, media ingredients, and culture media were of analytical grade (Hi-Media, India and Sigma-Aldrich, USA). The mineral salt medium (MSM) comprised (g *L*^−1^): K_2_HPO_4_ 1.0, KH_2_PO_4_ 1.0, (NH_4_)_2_.SO_4_ 2.0, CaCl_2_ 0.1, MgSO_4_ 0.2, MnSO_4_ 0.02, FeSO_4_ 0.05, and peptone 5.0.

### 2.2 Isolation and screening of ligninolytic bacterial isolates

Papermill sludge was collected from M/s Yash Paper Mill located at Ayodhya, Uttar Pradesh, India, and used for isolation of ligninolytic bacteria using the enrichment method. Enrichment of ligninolytic bacteria was done in MSM amended with 500 mg *L*^−1^ KL and 0.5% glucose at 30 °C for 5 days. The culture broth was serially diluted and spread on KL amended MSM plate and incubated at 32°C for 2 days to isolate the enriched ligninolytic bacteria. As a result of enrichment and preliminary screening, a total of seven bacterial colonies (LB1-LB7) were purified by repeated screening on KL-MSM agar plates as ligninolytic bacteria. The pure culture of the bacteria was maintained in glycerol stock and the working culture was stored at -20 °C. The lignin utilization potential of selected bacteria, as well as previously known ligninolytic bacteria such as *Serratia liquefaciens* [11], *Kocuria rosea* (MTCC 1532), and *Pseudomonas putida* (MTCC 7525), were tested by growing them in three different growth media: M1) MSM, M2) MSM+KL (500 mg *L*^−1^) and M3) MSM+KL (500 mg *L*^−1^) + glucose (0.5%). For this study, the inoculum was grown overnight (18 h) at 32 °C in Luria Bertani broth. The growth medium (10 mL) in the test tube (20 mL) was inoculated with 20 µL inoculums and incubated for 2 days at 32 °C. Afterward, culture broth (2 mL) was centrifuged (5000 rpm, 10 min) and the cell pellet was mixed with 2 mL of distilled water to measure the bacterial biomass at 600 nm. Following the Aneja, K. R. (2010) the biochemical characterization was performed.

### 2.3 Screening of selected bacterial isolates for ligninolytic enzyme

The selected bacterial isolates were screened for laccase, LiP, and MnP activity [4]. MSM agar plate was prepared by addition of guaiacol: 0.05%, Azure-B: 0.01%, and phenol red: 0.01% in MSM for the observation of laccase, LiP, and MnP activity, respectively. The plate was inoculated with one loopful overnight bacterial culture and incubated at 32 °C for 5 days. LiP and MnP activity of isolate was observed by the Azure-B and phenol red decolorization, respectively. The bacterial colony appeared as brown on the guaiacol-MSM agar plate, indicating laccase activity. Among seven isolates, only LB7 was capable to produce both LiP and MnP activity, hence LB7 was chosen for the antecedent study. This selected isolate was grown in MSM broth supplemented with Azure B and phenol red as the substrate for the quantification of LiP and MnP activity, respectively [11,42]. For determination of LiP and MnP activity, the MSM (100 mL, pH = 7.6) was added with 0.01% of Azure B and 0.01% phenol red dye, respectively after sterilization of the medium, and the media was inoculated with 1 mL of inoculum (OD_600nm_ = 0.6) and incubated for 5 days at 32 °C under 120 rpm shaking mode [11,42]. The quantification of LiP and MnP activity was determined in centrifuged supernatants taken at different time intervals. To measure the LiP activity, an enzymatic reaction mixture was prepared as per Riyadi et al. [27]. The mixture contained 1.0 mL of 100 mM sodium tartrate buffer (pH 3.8), 1.0 mL of 4 mM veratryl alcohol, and 0.2 mL of crude enzyme and was incubated at 32°C for 5 min. Thereafter, 0.2 mL H_2_O_2_ (2 mM) was added to trigger the reaction and absorbance changes were recorded at 310 nm by spectrophotometer (UV-1800, Shimadzu, Japan). Enzymatic reaction mixture to measure the MnP activity was comprised: 50 mM sodium tartrate buffer (pH 4.5) + 0.5 mM 2, 6-DMP (2, 6-dimethyl-phenol) + 0.2 mM MnSO_4_+ crude enzyme. H_2_O_2_ (0.1 mM) was added to initiate the reaction and the absorbance changes were recorded at 469 nm [14]. One international unit (IU) of the enzyme is defined as the amount of enzyme that oxidizes 1 µmol of substrate per minute. The enzyme activity was assayed in triplicate.

### 2.4 Bacterial identification

Molecular identification of LB7 isolate was carried out by a 16S rRNA gene sequencing method. Genomic DNA was PCR amplified for 16S rRNA gene using universal primers (27F: 5′-AGAGTTTGATCMTGGCTCAG-3′) and (1492R: 5′-GGTTACCTTGTTACGACTT-3′) in a PCR reaction mixture at standard PCR conditions (35 cycles): Denaturation at 94 °C (1 min), annealing at 45 °C (1 min) and followed by the extension at 72 °C (2 min). PCR product was purified by a QIA gel extraction kit. The PCR amplicons were probed with a Genetic Analyzer (ABI 3730xl). The gene sequencing was outsourced from M/s. Eurofins Genomics, Bangalore, India. With the available database at NCBI GeneBank (http://www.ncbi.nlm.nih.gov/), the 16S rRNA gene was used to BLAST. The sequences were selected based on the maximum similarity and aligned using ClustalW software. MEGA 7 was used for the construction of a phylogenetic tree [39]. The isolate was also tested for some morphological and biochemical characteristics [3].

### 2.5 KL biodegradation studies

Lignin biodegradation by LiP and MnP producing isolate was performed in conical flasks (500 mL) containing sterilized KL-MSM medium (100 mL) supplemented with glucose (0.5%). The 1% (v/v) inoculums were inoculated in the flask with pH 7.6 and incubated at 32°C in a shaker (Kuhner shaker X, USA) at 120 rpm. The study experimented in triplicate. The samples were harvested at 0 h and on 1,3,5,7 and 14 days of incubation. The sample was taken at 0 h and treated as the control. All the samples were centrifuged (5000 rpm, 10 min) and the supernatant was analyzed for KL degradation. Determination of bacterial growth in the culture medium was measured at OD at 600 nm as per the above-discussed method in Section 2.2. The KL content was determined spectrophotometrically at 280 nm [44].

### 2.6 KL degradation analysis

For this study, the bacterium was cultivated in MSM containing KL (500 mg *L*^−1^) for 14 days and un-inoculated KL-MSM served as a control. The cell-free supernatant was processed for chemical analysis.

#### 2.6.1 UV–Vis spectral analysis

The supernatants of control and bacterial degraded KL samples were filtered using a 0.45 µm PVDF membrane. The UV–Vis absorption spectra of the samples were taken between 250 and 500 nm on a spectrophotometer [1].

#### 2.6.2 SEM analysis

The residual KL from the supernatant was separated by the acid precipitation method using 12 M HCl [25]. The precipitates were dried and mounted on aluminum stabs coated with gold-palladium alloy in a MiniSputter Coater (Model SC7620, Quorum, Technologies, UK) and viewed in SEM (Quanta 450 FEG, FEI, Netherlands). The elemental analyses of the samples were done by energy-dispersive X-ray (EDX) spectroscopy.

#### 2.6.3 FTIR spectroscopy

The acid precipitated residual KL was dried and samples were loaded onto the diamond crystal of the FTIR spectrometer (Nicolet™ iSTM, USA). The IR spectra (4000–1000 cm^–1^) of each spectrum were obtained with a resolution of 4 cm^–1^ and 31 scan numbers.

#### 2.6.4 GC–MS analysis

Fifty-milliliter samples were centrifuged (8000 g, 20 min) to remove cell biomass. Concentrate HCL was used to maintain the pH (2.0) of supernatants and then extracted with ethyl acetate. The pooled samples were purged with sodium sulfate and passed through filter paper (Whatman no. 1) and evaporated at room temperature overnight. The residues were derivatized with trimethylsilyl [BSTFA N, O-bis(trimethylsilyl) trifluoroacetamide) and trimethylchlorosilane (TMCS)] [12]. Derivatized samples (1 µL) were analyzed on GC–MS (ThermoFisher Scientific, UK). The electron ionization mass spectra of metabolites were verified in the range of 30–550 (*m/z*) at 70 eV. The NIST library available with the equipment was used to identify the peaks.

### 2.7 Statistical analysis

All data are expressed as the mean ± standard deviation (SD) and GraphPad Prism 9.0 (GraphPad Software, USA) was used for statistical analyses.

## 3 Results and discussion

### 3.1 Isolation, screening, and characterization of ligninolytic bacteria

**Fig. 1:**
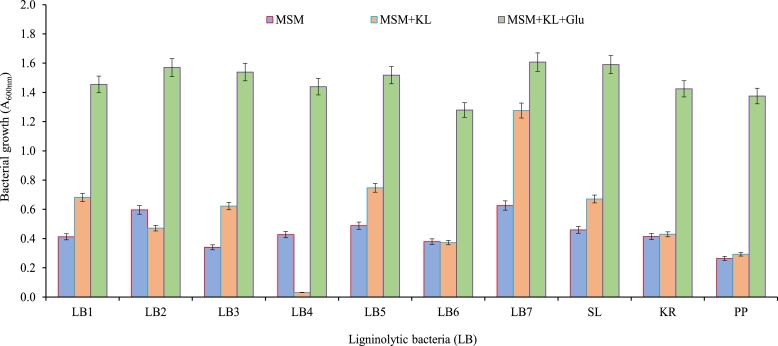
A screening test for selecting KL-utilizing bacteria. The preliminarily selected bacteria were grown in culture media (MSM, MSM+KL, and MSM+KL+Glu) for 2 days at 32 °C under shaking (120 rpm) and the OD of bacterial cells was measured at 600 nm. MSM = mineral salt medium, KL = kraft lignin (500 mg *L*^−1^), Glu = glucose (0.5%). SL = *Serratia liquefaciens,* KR = *Kocuria rosea,* and PP = *Pseudomonas putida* were included as standard ligninolytic bacteria.

Seven different ligninolytic bacteria (LB1-LB7) were initially isolated on the KL-MSM agar plate (500 mg *L*^−1^). The newly isolated ligninolytic bacteria, as well as previously known ligninolytic bacteria (*S. liquefaciens:* SL*, K. rosea:* KR, and *P. putida:* PP), were grown in MSM and MSM+KL added with and without glucose (0.5%) to test lignin utilization potential (Fig. 1). All bacteria exhibited growth in MSM. However, the addition of KL in MSM increased the growth of bacterial strains LB1, LB3, LB5, LB7, and *S. liquefaciens* indicating that KL can support the growth of isolates as a carbon source. Further, the addition of glucose (0.5%) to the KL+MSM medium enhanced all bacterial growth manifolds. The KL utilization rate of newly isolated bacteria was higher than the known ligninolytic bacteria (*S. liquefaciens, K. rosea* and *P. putida)* used in this study for comparison. Therefore, known ligninolytic bacterial strains were not included in subsequent studies. KL can be degraded by some potential fungi but it needs glucose as an energy source to digest the KL [16]. Bacteria also need another carbon source to initiate growth for KL degradation [8,35]. However, in the present study, isolated bacteria showed increased growth in MSM with a low concentration (0.5%) of glucose; needed for initial bacterial growth to metabolize KL. Whereas, KL was the only high concentration (500 mg/l) carbon source, which indicates the potential for KL to be used.

**Fig. 2:**
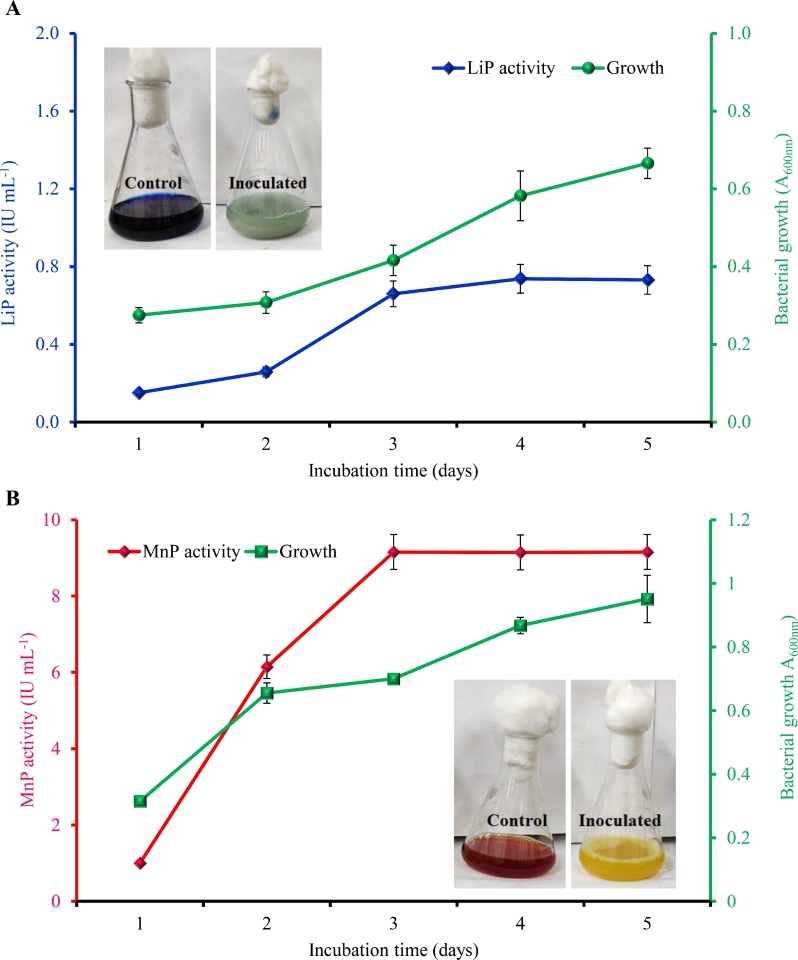
Quantification of ligninolytic enzymes from *B. aryabhattai* at different time intervals. Azure B and phenol red were used as indicator dyes for LiP and MnP activity, respectively at the final concentration of 0.01%. (a) LiP activity and (b) MnP activity.

The isolated seven ligninolytic bacteria were investigated for the presence of LiP, MnP, and laccase enzymes. Out of these seven bacterial isolates, only one bacterium (LB7) showed production of both LiP and MnP enzymes during growth in MSM broths added with Azure B and phenol red dye, respectively. Fig. 2a shows time-course LiP activity by the bacterium during Azure B dye decolorization (insert in Fig. 2a), which showed growth-associated enzyme production to be optimal (0.74 IU mL^−1^) on the 4th day. The result of the MnP production by the isolate in MSM broth is presented in Fig. 2b which also revealed the growth-associated production of MnP with simultaneous decolorization of phenol red (insert in Fig. 2b). The maximum bacterial growth (OD_600_ 1.0) and MnP activity (9.2 IU mL^−1^) were observed on the 4th and 5th day, respectively. No decolorization of Azure B and phenol red was observed in the control (uninoculated). Further, this isolate did not show laccase activity in the MSM-guaiacol broth medium. During the enzymatic KL degradation and decolorization of paper mill effluent, LiP and MnP has been reported as the key enzymes [6,11]. Baghel and Anandkumar [4] reported that ligninolytic enzymes are bacterial extracellular enzymes and are involved in KL degradation and detoxification. Lignin peroxidase mainly oxidized aromatic non-phenolic moiety of lignin structures, resulting in the formation of aryl-cation radicals. Whereas, manganese peroxide oxidizes the phenolic parts of the lignin structure to produce free radicals [40]. The maximum level of MnP (258.57 U *L*^−1^) and LiP (422.68 U *L*^−1^) activity was observed by *B. amyloliquefaciens* SL-7 on the 4th day of incubation [23]. Zainith et al. [42] reported that *B. aryabhattai* was able to produce 4.7 IU mL^−1^ manganese peroxidase activity within 72 h in 100 mg *L*^−1^ lignin-containing MSM broth.

During the enzyme (ligninolytic enzyme) mediated process, larger macromolecules (protein) interact with smaller molecules (signaling molecules), then form highly active intermediates, which can react with KL and cause the cleavage of chemical bonds in the KL structure [23]. Like ligninolytic enzyme laccase which oxidizes the phenolic hydroxyl of KL and unstable the aromatic ring of internal structure. Molecular characterization of this isolate was performed by 16S rRNA sequencing. The partial nucleotide sequence of LB7 was matched with the available database (https://www.ncbi.nlm.nih.gov/) using BLAST. It was revealed that LB7 showed maximum homology 99.80%, with *Bacillus aryabhattai* strain CCM 2010 (NR_115,953.1). Therefore, LB7 was identified as *B. aryabhattai* with accession number MW065484. The conventional tests for bacterial characterization indicated the isolate was non-motile, gram-positive, rod-shaped, fermentative, and showed a positive reaction of catalase. It was able to grow at 50 °C and tolerated 4% NaCl. The first time *B. aryabhattai* was isolated and identified from the air collector cryotubes used at a high altitude (27–41 km) (Shivaji et al. [33].

However, to date numbers of *B. aryabhattai* strains have been isolated from different sources such as plants, soil, and an urban tunnel [5]. Ligninolytic *B. aryabhattai* was also isolated from paper mill effluent wastewater and soil, straw, and sludge [41,42].

### 3.2 KL biodegradation

**Fig. 3:**
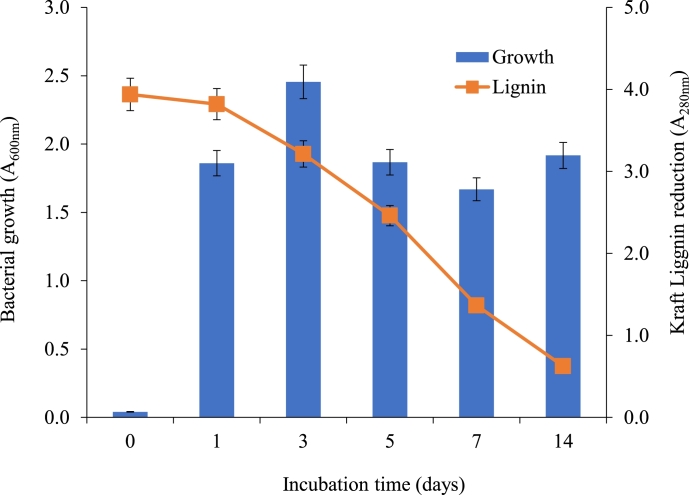
Growth and KL degradation by *B. aryabhattai* at a different time interval at 32 °C under shaking (120 rpm) in MSM containing 500 mg *L*^−1^ KL.

The ligninolytic *B. aryabhattai* KL degradation potential was determined with the absorbance at A_280nm_ on different days during 14 days of incubation with 500 mg *L*^−1^ KL-MSM supplemented with glucose (0.5%). Fig. 3 presents the time course of bacterial cell growth and KL degradation. Maximum bacterial growth (A_600nm_ = 2.46) was observed on the 3rd day, while KL degradation was started from day one and continued for 14 days. At the end of the experiment (14 days), the absorbance A_280nm_ was reduced from 2.94 to 0.46. The ligninolytic bacterial strain *B. aryabhattai* was capable to degrade KL up to 84% in 14 days of the incubation period. It is well known that KL cannot be metabolized by bacteria in the absence of an additional carbon source. In the present study, 0.5% glucose was added to MSM which supported initial bacterial growth and subsequent use of KL as a co-metabolism. Similar types of findings have been reported by several researchers [11,42]. The removal of 54% of lignin from paper mill wastewater by MnP producing *B. aryabhattai* was reported within 144 h of treatment at 32°C, pH = 7.6, and 120 rpm [42]. Mei et al. [23] has also reported that the bacterium *B. amyloliquefaciens* SL-7 was able to degrade 28.55% of lignin in tobacco straw within 15 days of treatment.

### 3.3 Confirmation of KL biodegradation by SEM-EDX

**Fig. 4:**
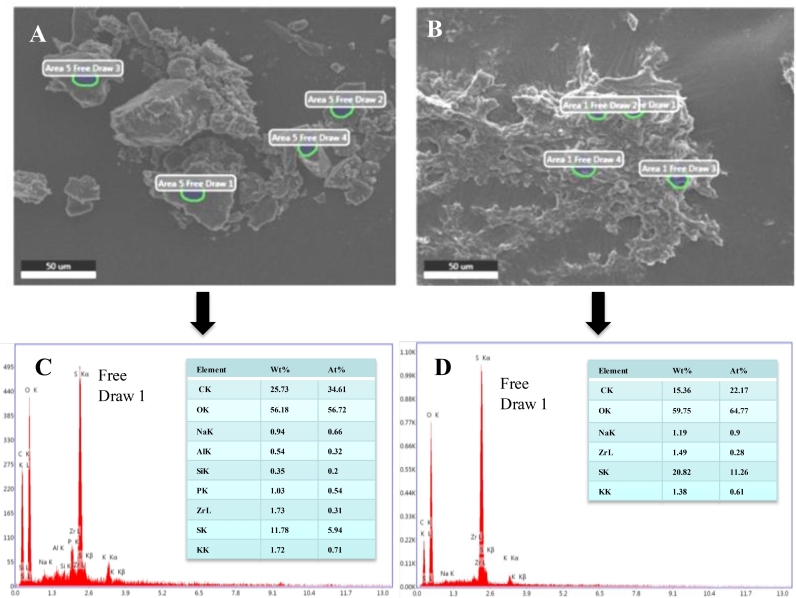
SEM images of KL before (a) and after degradation (b) by *B. aryabhattai* and corresponding EDX (c and d).

Surface morphology and structural changes in KL samples were analyzed and compared before and after bacterial degradation to confirm KL biodegradation. The scaling and magnification in all the SEM images were kept identical for comparative evaluation. The SEM images of KL samples before and after bacterial degradation showed different morphologies (Fig. 4a-b). It can be seen from Fig. 4a, that the KL sample before bacterial treatment showed a bigger particle size, and a flat smooth surface with crushed ends, whereas the SEM image of KL after bacterial degradation (Fig. 4b) showed a smaller particle size, crushed eroded surface with dawdle deposits. Results showed that the flat smooth surface of the KL sample was completely eroded and ruptured after bacterial degradation. Further, the KL structure was completely degraded and transformed, and their particle size was found to be reduced throughout bacterial degradation. The results indicated that the degradation and transformation of KL were mainly due to the involvement of *B. aryabhattai*. Similar observations on biodegradation and transformation of KL were reported by earlier researchers [18,35,44].

The elemental compositions of KL before and after bacterial degradation were compared to analyze the changes in their mineral composition. The results from EDX analysis indicated higher O, C, and S content as compared with other mineral contents present in KL before and after degradation. The O, Na, and S content of KL before treatment was 56.18, 0.94, 11.78 Wt%, and these mineral contents were increased after bacterial treatment. The higher O content indicated the availability of a high number of oxygen-containing functional groups as suggested by earlier researchers [26,29]. The Al, Si, and P content was not detected in KL after bacterial degradation. The results showed variation in mineral contents of KL as well as bacteria degraded KL due to degradation and structural transformation of KL facilitated by bacterial isolate.

### 3.4 UV–Visible spectroscopy

UV–Vis spectroscopy analysis is the basic technique frequently used by researchers to characterize KL degradation [1]. The UV–Vis spectral analysis of KL exhibited a strong peak between 250 and 280 nm before and after bacterial degradation, there was a decrease in absorbance of the original peak in the UV region of the spectral scan (Fig. S1). The decrease in absorbance of the peak indicates degradation of KL due to bacterial metabolic activity. This finding correlated with a previously reported study related to the UV–Vis spectrum observed during the lignin and chlorolignin degradation of paper mill wastewater by *Bacillus* sp. IITRDVM-5 [36]. Another study [17], was also reported a declining trend in the UV–Vis spectrum of *B. cereus* strain AKRC03 treated paper mill wastewater.

### 3.5 FTIR spectroscopy

**Fig. 5:**
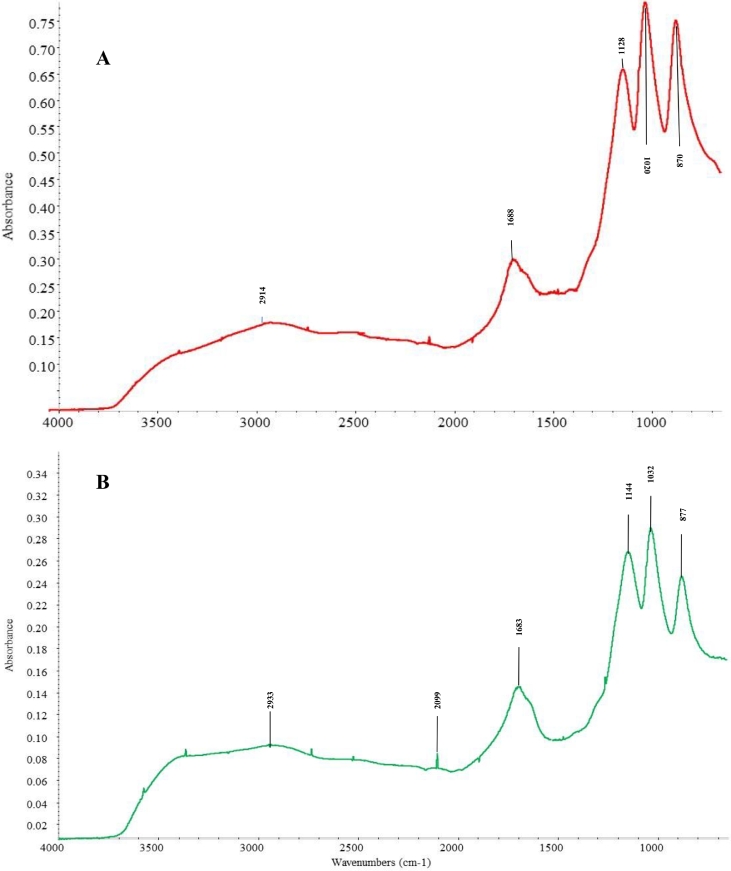
FTIR spectra of KL before (a) and after (b) degradation by *B. aryabhattai* (14 days)*.*

The IR spectra (4000–1000 cm^−1^) of KL before and after bacterial degradation were compared to detect chemical changes on lignin structure. Results showed several strong and prominent IR absorption peaks at different wavenumbers 2914, 1688, 1128, 1020, and 870 cm^−1^ were assigned to various functional groups are present in KL (Fig. 5a-b). The absorbance of these peaks were observed to decreased after bacterial treatment which indicate biodegrdation of KL by *B. aryabhattai*. The IR absorption peaks recorded in IR spectra of KL samples at around 2914–2933 cm^−1^ correspond to C–H stretching vibration in CH_3_O, CH_3_, and CH_2_ functional groups of KL [38]. The IR absorption peaks of the bonds at their respective corresponding location as described above were found to increase in bacterial degraded KL as compared with the non-degraded KL which indicated change in the methyl group [21]. The peaks observed at around 1683–1688 cm^−1^ correspond to the C=O stretch of the carbonyl group and absorbance intensity of this peak was substantially reduced (∼45%) after biodegradation indicating breakage/replacing aromatic ring chain reactions [21]. These results indicated that their reduction of benzene ring structural units might happen during KL biodegradation. The IR absorption peak at around 1128 cm^−1^ corresponds to –O– and C–O asymmetrical stretching vibration in the ether linkage and carboxylic group, respectively, which indicate side-chain oxidation and demethylation in KL structure [21]. Further, the peaks that appeared at around 1020 and 870 cm^−1^ are assigned to the C–N stretch in–the NH_2_ group and aromatic C–H stretch, respectively. On comparing different IR peaks in the corresponding curve of each sample, the overall results of FTIR analysis concluded that there was substantial changes in KL structure due to demethylation, cleavage of various ether linkages, and oxidation during KL biodegradation.

### 3.6 GC–MS analysis

**Fig. 6:**
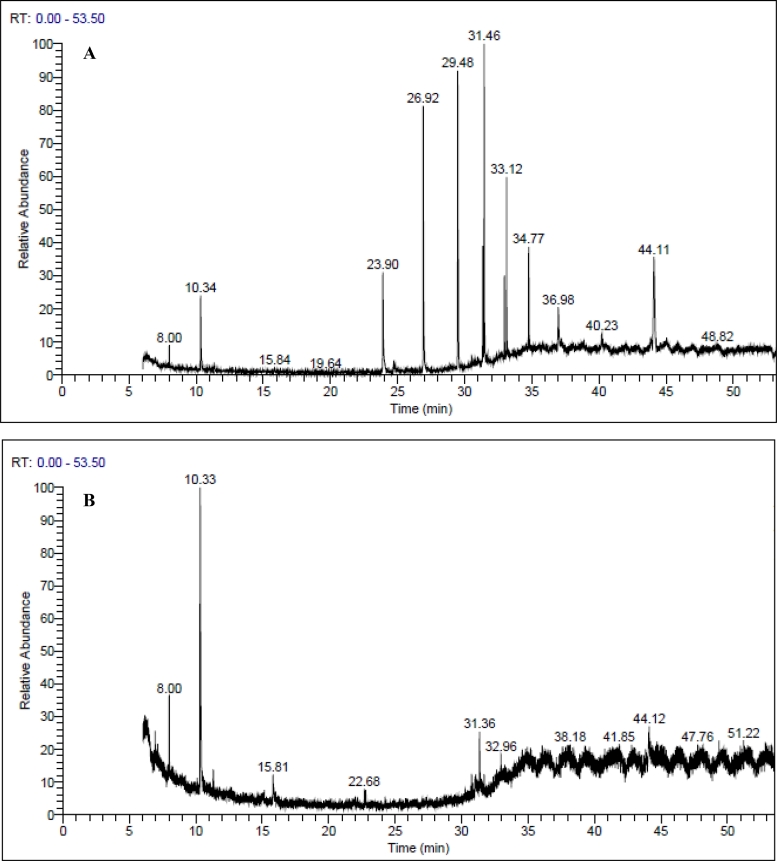
GC–MS chromatogram of ethyl acetate extracts of control KL (a) and KL degraded with *B. aryabhattai* after 14 days. The MS-identified compounds with respect to their retention are listed in Table 1.

GC–MS analysis was performed for control and bacterial treated sample to monitor KL degradation metabolites. GC–MS has been proven as a powerful tool to confirm the ability of bacteria to degrade KL [32,35]. The GC–MS total ion chromatogram (TIC) of ethyl extract of control and bacterial degraded samples is shown in Fig .6(a,b), and their peak identity is illustrated in Table-1. The result shows the appearance and disappearance of peaks in the TIC of samples. Most of the GC–MS identified compounds [2,3-bis (trimethylsilyl)−1,4-diphenylbutane (RT = 8.00), 3-(4-hydroxyphenyl)−2-methyl-2-(naphthalen-2-ylsulfonylamino) propanoic acid (RT = 23.90), 3-(benzyloxycarbonyl)−1-(p-toluenesulfonyl)−5,6-dihydro-2(1H)−2-pyridone (RT = 26.92), 2,5-dimethoxy-N-(4-henoxyphenyl) benzenesulfonamide (RT = 31.46), Docosanoic acid, 1,2,3-propanetriyl ester (RT = 33.12), 3-(benzyloxycarbonyl)−1-(p-toluenesulfonyl)−5,6-dihydro-2(1H)−2-pyridone (RT = 34.77), 3-(benzyloxycarbonyl)−1-(p-toluenesulfonyl)−5,6-dihydro-2(1H)−2-pyridone (RT = 36.98), 3‑tert‑butyl‑5-methyl-4-hexen-2-ol (RT = 44.10)] in control were eliminated during the bacterial degradation of KL after 14 days. The TIC of the bacterial degraded KL sample showed the formation of metabolites during the bacterial KL degradation process which were identified as diisopropylidene mannitol (RT = 15.81), Ethyl 4, 4, 4-trichloro-1-butenyl carbonate (RT = 31.36) and (2R, 3S)−1, 2-epoxy-4-penten-3-ol (RT = 44.12). The identified metabolites were not related to the low molecular weight unit of the KL structure. KL degradation is a biological process that involved carbon atoms reduction which leads to a decrease in its molecular weight. It comprises both incomplete degradations of larger molecules into smaller phenolic compounds [43,44], and the complete degradation of larger KL molecules into CO_2_ and H_2_O. In the present study, metabolites identified by GC–MS were not related to the low molecular weight unit of the KL structure. The result may be due to the low content of phenolic compounds that were not detected by GC–MS.

**Table 1:** A compound identified as trimethylsilyl (TMS) derivatives in ethyl extract from control and bacterial degraded KL samples is given in Fig. 6.

RT (min)	NIST-identified compounds	Fig. 6a	Fig. 6b
8.00	2,3-bis (trimethylsilyl)−1,4-diphenylbutane	+	+
15.81	Diisopropylidene mannitol	–	+
23.90	3-(4-hydroxyphenyl)−2-methyl-2-(naphthalen-2-ylsulfonylamino) propanoic acid	+	–
26.92	3-(benzyloxycarbonyl)−1-(p-toluenesulfonyl)−5,6-dihydro-2(1H)−2-pyridone	+	–
31.36	Ethyl 4,4,4-trichloro-1-butenyl carbonate	–	+
31.46	2,5-dimethoxy-N-(4-phenoxyphenyl)benzenesulfonamide	+	–
33.12	Docosanoic acid, 1,2,3-propanetriyl ester	+	–
34.77	3-(benzyloxycarbonyl)−1-(p-toluenesulfonyl)−5,6-dihyd ro-2(1H)−2-pyridone	+	–
36.98	3-(benzyloxycarbonyl)−1-(p-toluenesulfonyl)−5,6-dihydro-2(1H)−2-pyridone	+	–
44.10	3‑tert‑butyl‑5-methyl-4-hexen-2-ol	+	–
44.12	(2R,3S)−1,2-epoxy-4-penten-3-ol	–	+

-TMS derivatives of ethyl acetate extract from control (Fig. 6a) and degraded by *B. aryabhattai* after 14 days (Fig. 6b).

## 4 Conclusion

The ligninolytic enzyme LiP and MnP enzyme-producing bacterium *B. aryabhattai* was able to degrade KL which was established through analysis of the degraded KL samples using SEM, FTIR, and GC–MS. Among all the ligninolytic enzymes MnP showed maximum activity (9.2 IU mL^−1^) by the *B. aryabhattai* The optimum KL reduction (84%) was observed in MSM with 0.5% glucose at 32°C, pH 7.6, and 120 rpm. SEM analysis showed that the smooth surface of KL was eroded after bacterial degradation. FTIR results indicated that the reduction of benzene ring structural units might have happened during KL biodegradation. GC–MS analysis revealed that compounds identified in ethyl extracts of control KL were eliminated after bacterial degradation. Ligninolytic *B. aryabhattai* provides a basis for potential KL biodegradation and may be used for the treatment of other lignin-containing industrial effluents. However, further studies are needed to fully understand the metabolic characteristics of the KL and the degradation process by *B. aryabhattai*.

## Author contribution statement

**Anjali Singh:** Qualitative and quantitative analysis of ligninolytic enzymes, sample preparation for GC–MS and SEM-EDX, writing-original draft. **Rajesh Kumar:** Bacterial isolation, lignin degradation experiments, sample preparation for UV–Vis and FTIR. **Annapurna Maurya**: Data analysis, software supervision. **Pankaj Chowdhary:** Writing-review, and editing of the manuscript. **Abhay Raj**: Supervision, conceptualization, data analysis, writing-review, and editing of the manuscript.

## Declaration of Competing Interest

The authors declare that they have no conflict of interest.

## Appendix Supplementary materials

Image, application 1
