# Supplementary material for: Isolation of functional ligninolytic Bacillus aryabhattai from paper mill sludge and its lignin degradation potential
Source: Biotechnol Rep (Amst). 2022 Jul 18;35:e00755. doi: 10.1016/j.btre.2022.e00755 (PMC9307452; doi:10.1016/j.btre.2022.e00755)
Supplement: Supplementary file 1 [file mmc1.doc]

**Supplementary Information**

**Fig. S1:** Ultraviolet-visible (UV-Vis) analysis of KL before (a) and after (b) degradation by *B. aryabhattai* (14 days)*.*
